# NAVIGATING FINANCIAL CONVERSATIONS ABOUT STUDENT LOAN DEBT AMONG OLDER BORROWERS AND THEIR FAMILIES

**DOI:** 10.1093/geroni/igz038.1099

**Published:** 2019-11-08

**Authors:** Samantha Brady, Julie Miller, Alexa Balmuth, Lisa D'Ambrosio, and Joseph Coughlin

**Affiliations:** MIT AgeLab, Cambridge, Massachusetts, United States

## Abstract

Currently, approximately 44 million people in the United States carry the weight of over 1.4 trillion dollars of student loan debt. As the cost of education continues to rise, the decision of taking on student loans is increasingly a family decision rather than an individual one. While the majority of research focuses on younger borrowers, little research has been done to understand the experiences of parents and grandparents taking on student loans for a loved one. In order to financially and emotionally manage this burden, borrowers may benefit from support from their social networks, including family and friends. For many, navigating these difficult conversations presents a challenge of its own. This presentation will spotlight an MIT AgeLab mixed methods study about how student loan borrowers between the ages of 51 and 75 experience and manage their student loans within family systems and how these loans may impact family dynamics. Data were collected through focus groups and a national survey. Preliminary findings suggest that older borrowers demonstrate several distinct communication typologies within their families in regards to finances, particularly regarding student loan accrual and repayment. Each of the four primary communication styles regarding loans impact borrowers’ financial and emotional wellbeing throughout the life course, as well as perceived relationship dynamics. Moreover, older borrowers are more likely to report family conflict if student loans are less frequently discussed with family members. Findings also suggest strategies to help parents and grandparents facilitate conversations abound student loans based on their unique family communication styles.

